# Assembling Composite Dermal Papilla Spheres with Adipose-derived Stem Cells to Enhance Hair Follicle Induction

**DOI:** 10.1038/srep26436

**Published:** 2016-05-23

**Authors:** Chin-Fu Huang, Ya-Ju Chang, Yuan-Yu Hsueh, Chia-Wei Huang, Duo-Hsiang Wang, Tzu-Chieh Huang, Yi-Ting Wu, Fong-Chin Su, Michael Hughes, Cheng-Ming Chuong, Chia-Ching Wu

**Affiliations:** 1 Department of Biomedical Engineering, National Cheng Kung University, Tainan, 701, Taiwan; 2 Institute of Basic Medical Science, National Cheng Kung University, Tainan, 701, Taiwan; 3 Division of Plastic Surgery, National Cheng Kung University Hospital, Tainan, 701, Taiwan; 4 Department of Cell Biology and Anatomy, National Cheng Kung University, Tainan, 701, Taiwan; 5 International Research Center for Wound Repair and Regeneration, National Cheng Kung University, Tainan, 701, Taiwan; 6 Institute of Clinical Medicine, National Cheng Kung University, Tainan, 701, Taiwan; 7 Department of Pathology, University of Southern California, California 90033, USA

## Abstract

Intradermal adipose tissue plays an essential role for hair follicles (HFs) regeneration by regulating hair cycles. However, the effect of reconstruction of HFs and the involvement of adipose-related cells are poorly understood. We investigated assembly strategies for the interactions of dermal papilla (DP) cells with adipose-derived stem cells (ASCs) in promoting hair formation. DP cells lose DP traits during adherent culture, but preserved DP markers with a unified sphere diameter by seeding on chitosan-coated microenvironments. Next, ASCs isolated from rats were co-cultured with DP spheres by different assembling approaches to determine their interactions; a mixed sphere of ASCs with DP cells (MA-DPS), or a core-shell structure, outer ASCs shell and an inner DP core (CSA-DPS). CSA-DPS exhibited superior DP characteristics compared to MA-DPS. Conditional medium from ASCs, but not differentiated adipocytes, promoted DP markers and functional alkaline phosphatase activity from the DP cells. *In vivo* patch assay showed the core-shell assembling of CSA-DPS can reconstruct cellular arrangements and microenvironmental niches as dominated by PPARα signal in ASCs to induce the greater hair induction than MA-DPS or DP spheres alone. Therefore, the assembling of a core-shell sphere for DP with ASCs could reconstruct the HF cellular arrangement for hair formation. This paper set the groundwork for further evaluation of the input of other cell types.

Hair loss or alopecia is associated with aging or hormonal perturbance in males and females that results in the loss of follicular stem cell activity to form hair germs for hair follicle regeneration^1^. Hair loss also occurs due to the loss of full-thickness skin regions in severe wounding or burns from accidents. Currently, medication and hair transplantation are two major treatments for hair loss^2^,^3^,^4^. Therapeutic drugs, such as finasteride, dutasteride, and minoxidil mainly function by preventing further hair loss rather than rebuilding lost hair^3^. Although the transplantation of hair is a minimal invasive surgery to move healthy hair follicles (HFs) to the bald area, the number of hairs is not sufficient in patients with severe hair loss.

The activity of hair stem cells can be influenced by the microenvironment inside the HFs or the macroenvironmnet outside the HFs. The mature HF is a complex structure which contains several concentric epithelial cylinders of keratinocytes and a specialized mesenchyme of dermal papilla (DP) cells. The DP consists of a group of highly active specialized fibroblasts derived from the dermal mesenchyme and is a spheroid structure at the base of the HF that induces hair neogenesis^5^. The spheroid-like DP is believed to play a key role in hair cycling^5^ and serve as the physical niche for providing signals to matrix progenitors in specifying the size, shape, and pigmentation of hair fiber^6^,^7^,^8^.

Tissue engineering and regenerative medicine approaches identified several interactions between epithelial and dermal cells for HF morphogenesis and maintenance^9^,^10^. Some cells have potential hair-inductive capacity, including DP cells, dermal sheath cells, skin-derived precursor cells, and mesenchymal stem cells (MSCs)^11^. The hair inductive capacity can be further enhanced by activating specific signaling pathways and by DP cell aggregation^11^. DP cells shows aggregative behavior and this prolongs expression of DP specific markers when cultured in a three-dimensional (3D) environment by hanging drop or biomaterial culture systems, such as on hydrophilic polyvinyl alcohol (PVA)^12^,^13^,^14^. The aggregation of cultured DP cells into spheres enhances their hair-inductive capacity^15^,^16^,^17^,^18^,^19^,^20^. Chitosan, a natural component of chitin, is a linear polysaccharide composed of randomly distributed β-(1–4)-linked D-glucosamine (deacetylated) and N-acetyl-D-glucosamine (acetylated) units. It is ideal for biomedical applications because of the inherent biological properties of biocompatibility and biodegradability. Furthermore, chitosan as a surface coating substrate facilitates cell sphere formation in osteoblasts^21^, keratinocytes^22^ and hepatocytes^23^, as well as adipose-derived stem cells (ASCs)^24^.

The intradermal adipose tissue plays an important role in hair biology due to its expansion during the anagen phase resulting in an increase of adipocyte precursor cells^25^. During the telogen-to-anagen transition, adipocyte progenitor cells are activated to proliferate and form mature adipocytes surrounding the regenerating HF^26^. This outer layer of MSC-like cells surrounding the DPs may optimize the microenvironment to promote hair growth. ASCs possess similar multipotency as bone marrow MSCs, is obtainable in large quantities by liposuction, and can be an ideal source of unique autologous multipotency adult stem cells^27^,^28^. Peroxisome proliferator-activated receptors (PPAR) are ligand-activated transcription factors with three isoforms (PPARα, PPARγ, and PPARδ) and are all expressed in dermal and epithelial hair follicle cells. However, the roles of different adipose-related cells in HF formation or regeneration and tissue engineering are still unknown, especially in the contribution of PPAR signaling from ASCs or mature adipocytes. In this study, we are interested in testing the assembling of a HF-like structure with DP spheroids and the interaction of adipose-related cells to promote hair-like structure and regeneration. The underlying molecular mechanism for ASCs to benefit DP spheres is discovered via PPARα signaling in organized core-shell structures similar to the native HF.

## Results

### Preservation of dermal papillae characteristics with unified sphere size

**Figure 1:**
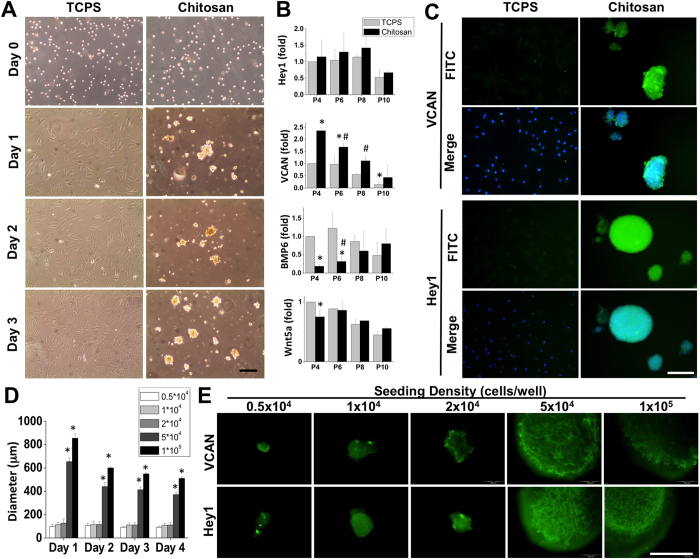
Dermal papilla (DP, 1.3 × 10^6^ cells) cells were seeded on tissue culture polystyrene dish (TCPS) or chitosan-coated microenvironment (CCM) for 10-cm dish (Chitosan) to observe the sphere formation by acquiring the phase images at Day 0, 1, 2, and 3 (**A**). The adherent culture of DP cells caused the loss of DP characteristic, versican (VCAN), after multiple passages while the sphere formation maintained the DP markers, such as VCAN and Hey1 (**B**). The induction of VCAN and Hey1 protein expressions were confirmed by immunofluorescent staining by double staining with DAPI for cells seeded on TCPS and Chitosan coated dishes (**C**). Although the chitosan-coated dish maintained the DP characteristics, high variability of sphere size and protein expression patterns were observed in both VCAN and Hey1 staining. To control the sphere size, the CCM was created on the 96-well culture plate and seeding cell with different density for each well for measuring the DP sphere size at Day 1, 2, 3, and 4 (**D**). When the seeding density exceeded 5 × 10^4^ cells per well, the DP cells formed a single sphere in each well and the sphere condensed during culture. The immunofluorescent staining of VCAN and Hey1 indicated the sphere derived from a higher seeding density than 5 × 10^4^ cells promoted better DP characteristics (**E**). *In (**B**) significant difference from the adherent DP cells at passage 4, p < 0.05. ^#^In (**B**) significant difference from the adherent DP cells at same passage, p < 0.05. *In (**D**) significant difference from seeding 0.5 × 10^4^ cells, p < 0.05. Scale bar = 200 μm.

When seeding rat vibrissal HFs DP cells on chitosan-coated TCPS dish, the cells exhibited aggregation with irregular morphology at Day 1 and formed floating spheroid-like structures on Day 3 (Fig. 1A). The regular culture of DP cells on a 2D adherent surface caused the gradual loss of DP characteristics between passage 4 and 10, especially for VCAN expression (P4~P10, Fig. S1). The semi-quantification results of gene expressions were normalized to passage 4 DP cells cultured on TCPS. On the other hand, the formation of DP spheres on chitosan-coated microenvironment preserved DP markers, such as VCAN and Hey1 gene expressions, and suggested the DP sphere can maintain the hair-inductive capacity of DP cells (Fig. 1B). During high passage of DP cells (P10), the Wnt/β-catenin signaling for Wnt5a expression was abolished in 2D culture, but was maintained during 3D DP sphere formation. The protein expressions of DP markers in spheres were confirmed by immunofluorescent staining of VCAN and Hey 1 (Fig. 1C). VCAN, a lectin protein family, was induced in DP spheres after seeding the cells in a chitosan-coated microenvironment. The expression of Hey1, a downstream target of Notch signaling, was increased after sphere formation. However, both of the fluorescent intensities from the VCAN and Hey1 IHC samples vary according to DP spheres size in 10-cm TCPS dish (Fig. 1C). By measuring the mean fluorescent intensities of spheres with different size, the positive correlations were observed between sphere size and the protein expressions of VCAN (R-square = 0.72) and Hey1 (R-square = 0.97) (Fig. S2). This indicates the regulation of DP characteristics may also be influenced by sphere size.

To create a uniform sphere size for the modulation of DP characteristics and hair regeneration, the formation of DP spheres with different diameters was tested by seeding the DP cells with increasing densities in chitosan-coated 96-well plates (Fig. S3). By controlling the seeding density, the sphere size can be unified in chitosan-coated 96-well plate. When the seeding density exceeds 5 × 10^4^ cells, the coating of chitosan on the bottom and side wall of 96-well promoted a quick aggregation of DP spheres on Day 1 with a loose spheroid structure, and then became a compact circular sphere after culturing for 4 days. The diameters of spheres with different seeding density were quantified by phase contrast images at different time points. After seeding the DP cells in chitosan-coated microenvironments for 3 days, the loading of 5 × 10^4^ cells in each well produced a sphere size with an approximate diameter of 400 μm, and 1 × 10^5^ DP cells will produce a sphere with an approximate diameter of 550 μm (Fig. 1D). The protein expressions of VCAN and Hey1 were increased in the large DP spheres compared to the small spheres formed by the low seeding density (Fig. 1E). There was no significant difference for the expression levels of VCAN and Hey1 between the seeding densities of 5 × 10^4^ and 1 × 10^5^ cells (Fig. S4). Therefore, the optimize loading density for DP cells in 96-well plate was chosen as 5 × 10^4^ cells according to the protein expression patterns and DP diameter.

### Different strategies of DP sphere assembly with ASCs

**Figure 2:**
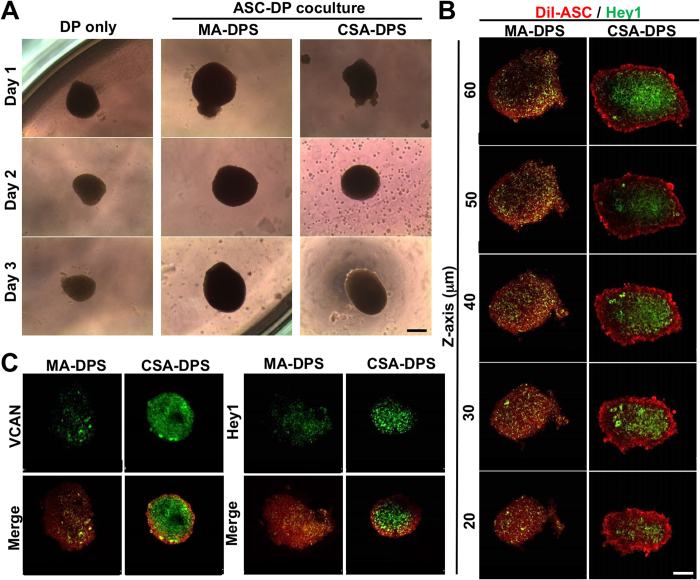
Assembling of adipose-derived stem cells (ASC) with DP cells was developed by; mixing ASC with DP cells (MA-DPS) for co-culture or sequential seeding of DP cells and then ASC to form a core-shell sphere (CSA-DPS) in 96-well CCM (**A**). In the phase images of CSA-DPS, the ASCs were added after the DP sphere formed at Day 2 and can assembled outside the DP sphere at Day 3. The ASCs was labeled with DiI (red color) to visualize the cell arrangement with immunofluorescent staining of Hey 1 (green color) after assembling for 3 days. The confocal images from different Z sections revealed the cellular arrangements in MA-DPS and CSA-DPS (**B**). The core-shell culture of ASCs with DP cells (CSA-DPS) enhanced the expressions of VCAN and Hey1, but the mixed culture decreased the expression of DP markers in MA-DPS (**C**). Scale bar = 100 μm

The adipose tissue surrounding HFs provides important signals for hair formation and growth. To understand how adipose-related cells may influence the DP sphere in spatial distribution, the rat ASCs were added at different assembling time points to form the MA-DPS or CSA-DPS with the same total cell numbers (Fig. 2A). The phase images demonstrated the chitosan microenvironment can promote sphere formation for both assembling methods. Loading of ASCs on the second day (phase image of CSA-DPS on Day2) achieved the sequential assembling of ASCs to the core sphere that formed by DP cells on Day1 (Fig. 2A).

The ASCs were labeled with DiI (red fluorescent dye) to reveal the spatial distribution of ASCs on the outer shell cellular arrangement of the sphere (Fig. 2B). In MA-DPS, serial scanning of confocal images in the Z-axis demonstrated a homogenous mixture of DiI-ASCs with DP cells throughout the sphere (Fig. 2B). For CSA-DPS, the immunefluorescent staining of Hey1 revealed a core structure of DP cells surrounded by an outer layer of DiI-ASCs at all Z section levels (Fig. 2B). VCAN and Hey1 protein expressions were increased in CSA-DPS compared to MA-DPS (Fig. 2C). The quantifications of fluorescent intensities confirmed the induction of VCAN and Hey1 by using CSA-DPS assembling (Fig. S5). This suggested a superior assembling of ASCs with the DP sphere may be achieved by using the core-shell structure.

### ASCs, but not adipocytes, promote DP characteristics

**Figure 3:**
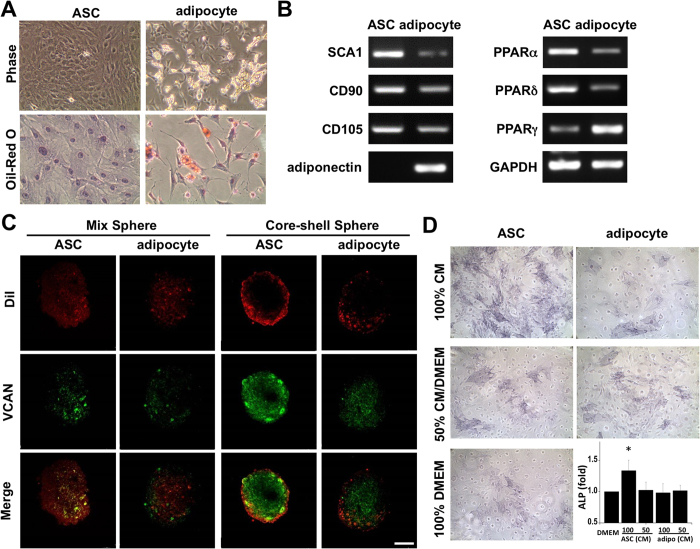
The mature adipocyte was differentiated from the same batch of ASCs by adipogenic induction medium. Significant accumulation of oil droplets was observed in adipocytes under phase image and confirmed by specific Oil-Red O staining (**A**). The induction of ASCs into mature adipocytes decreased the gene expressions of CD34 and SCA1, PPARα, and PPARδ (**B**). The mature adipocyte genes PPARγ and adiponectin were measured. The mature adipocyte can assemble into mix and core-shell spheres, but the expression of VCAN was further decreased compared to the co-culture of DP cells with ASCs (**C**). The conditioned media (CM) from ASCs increased the ALP activity compared to the culture of DP cells with fresh DMEM or CM from mature adipocyte (**D**). *Significant difference from 100% fresh DMEM, p < 0.05.

In subcutaneous adipose tissue, ASCs are interspersed amongst mature adipocytes. We further aimed to distinguish the difference between ASCs or mature adipocytes interacting with DP spheres. Lipid droplets were observed in the differentiated mature adipocytes, but not in ASCs, demonstrated by phase images (Fig. 3A, phase) and Oil Red O staining for the accumulation of lipid and fat (Fig. 3A, Oil Red O). The loss of stem cell markers, such as SCA1, was observed in differentiated adipocytes after adipogenic differentiation (Fig. 3B). The expressions of PPARα and PPARδ were decreased in mature adipocytes. On the other hand, increases of adipose gene specific expression of PPARγ and adiponectin were observed to indicate successful differentiation of mature adipocytes (Fig. 3B). Similar induction results were also observed in the ASCs isolated from human lipoaspirates and the resulting differentiated mature adipocytes (Fig. S6). The differentiated mature adipocytes induced from rat ASCs can also form a mix or core-shell structure with DP cells. Unlike the induction of DP markers in DP spheres using ASCs, both the mix and core-shell assembling of mature adipocytes with DP spheres showed inhibition of VCAN expression (Fig. 3C). The paracrine effects of ASCs and adipocytes on DP functions were tested by culturing the DP cells in different compositions of CM for 7 days and then assessed the ALP activity in DP cells to indicate cellular functions. The CM collected from ASCs (ASC, 100% CM) showed significant increase of ALP activity in DP cells compared to DP cells cultured in fresh DMEM (100%DMEM) or 50% CM/DMEM (Fig. 3D). The application of CM from mature adipocyte demonstrated no beneficial effect on ALP activity and was inhibited in DP cells. These results suggested that although the ASCs and adipocytes are both located in adipose tissue, their interaction and inductive function with DP cells are different. The ASCs are superior to mature adipocyte in maintenance of DP characteristics.

### Core-shell sphere patterning hair-like structure

**Figure 4:**
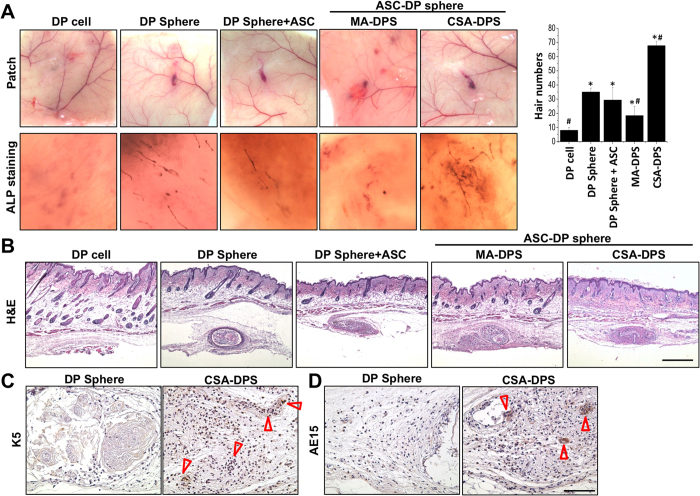
Hair induction was tested by *in vivo* patch assay in nude mice using suspending DP cells, DP spheres, DP spheres with suspending ASCs (DP Sphere + ASC), ASC and DP mixed spheres (MA-DPS), and the core-shell spheres with DP core and ASC shell (CSA-DPS). The CSA-DPS showed a highly pigmented patch, high ALP staining in whole mount tissue, and the most vivid hair number after 4 weeks of injections (**A**). H&E staining showed the success of hair follicle neogenesis in hypodermis (**B**). Distributions of brown color in immunohistochemistry (IHC) staining indicated expression of K5 for the outer root sheath of HF (arrow heads) (**C**). To confirm the hair-like structure in CSA-DPS, the inner root sheath was also identified by AE15 staining (**D**). *Significant difference from suspending DP cells, p < 0.05. ^#^Significant difference from DP sphere, p < 0.05. Scale bar in (**B**) = 500 μm. Scale bar in (**B**) = 100 μm.

The induction of hair-like structure was confirmed by *in vivo* patch assay using multiple sphere and cell combinations. Different groups of cells (different assemblies with or without ASCs) were mixed with single-cell suspended primary keratinocytes (KCs) and then injected into the hypodermis of nude mice. After injection for 4 weeks, the tissue patch was observed by harvesting the full-thickness skin and showed significant clusters of hair-like structures in the hypodermis of nude mice (Fig. 4A, patch image). The pigmentation and the HF-like structure were significantly increased in CSA-DPS group when compared to other groups. ALP staining was strong with the injected CSA-DP spheres but not for the mixed sphere of ASCs and DP cells (MA-DPS). Among the various combinations, the core-shell structure of DP core and ASCs shell (CSA-DPS) showed the greatest enhanced induction efficiency for hair-like structure formation (Fig. 4A, ALP staining). Quantification of the hair-like structures also indicates a significant improvement of hair induction when using the CSA-DPS assembling approach.

H&E staining revealed the structure of hair-like formation in the patch assay (Fig. 4B). The injection of single-cell suspended DP cells caused a cyst formation in hypodermis without any sign of hair induction or cluster of DPs. The application of DP spheres with different assembly structures of ASCs influenced the induction of hair formation. Specifically, we observed a significant induction of hair-like structures with the CSA-DPS spheres (Fig. 4B). This suggests the 3D arrangement of ASCs outside the DP spheres can promote the induction of hair-like structures in living animals. The contribution of transplanted ASCs were traced by labelling the ASCs with DiI and visualized in fluorescent image (Fig. S7). Transplantation of ASCs in different assemble strategies can deliver the ASCs in patch and showed different cell distributions. The evidence for the benefits of spatial arrangement was further confirmed by specific K5 staining for the outer root sheath of HF (Fig. 4C) and AE15 staining for the inner root sheath and medulla (Fig. 4D) in the hypodermis of CSA-DPS (brown positive staining in the enlarged images). A comparison of relative induction in CSA-DPS was examined with the positive reference of injecting the KCs with neonatal epithelial cells (Fig. S8). Although the CSA-DPS assembling cannot achieved best hair formation as neonatal cells, the superior cell arrangements and increases of HF markers still demonstrate beneficial concepts of CSA-DPS.

### PPARα mediate the enhancing effects of adipose tissue derived cells

**Figure 5:**
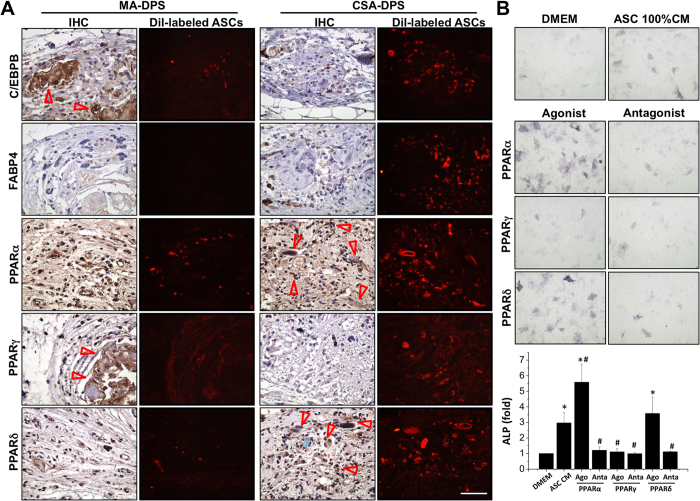
The IHC staining revealed the maturation of transplanted ASCs and the distribution of PPAR signaling during hair induction in the DP sphere, MA-DPS, and CSA-DPS (**A**). The transplanted ASCs (labeled by DiI) are distributed near the hair-like structure, but showed negative staining of early (C/EBPB) and mature (FABP4) adipocyte markers in CSA-DPS. The expressions of PPARα and PPARδ around the hair follicle (arrows) are important in hair-like structure and can only be observed in the CSA-DPS. PPARγ, which should be decreased in the hair structure, was highly expressed in the cell cluster of MA-DPS (arrows). The agonists and antagonists of different PPARs were applied to ASCs for modulating specific PPAR signal for 2 days and then replaced to fresh media for additional 2 days to collect the CM for DP cells (**B**). Increases of ALP activities in DP cells were observed when using the CM from ASCs with boost up PPARα signaling. On the other hand, the inhibitions of PPARα and PPARδ by specific antagonists abolished the beneficial effects in their CM. *Significant difference from DMEM, p < 0.05. ^#^Significant difference from ASC CM, p < 0.05. Scale bar = 50 μm.

We delved deeper into the PPAR signaling of these patch samples with the reference of a positive control. Among the different PPAR signals, PPARα and PPARδ were expressed in the outer layer of the HF-like cluster during hair induction (Fig. S9, arrows in PPARα and PPARδ staining). The CSA-DPS showed high agreement of similar cell arrangements and PPAR expression patterns with positive control (Fig. S9) that contrary to the MA-DPS (Fig. 5A). By tracing the DiI-labeled ASCs, the negative staining of early (C/EBPB) and mature adipocyte (FABP4) markers indicate the maintenance of stem cell properties in the ASCs when using the CSA-DPS assembly. In addition, the PPARγ did not express in the HF-like structures and expressed low intensity in the surrounding cells for CSA-DPS. However, the MA-DPS showed high C/EBPB and PPARγ indicates the mixture of ASCs with DP cells during the sphere formation might induce adipogenesis in the patch microenvironment (Fig. 5A, arrows in C/EBPB and PPARγ staining). To further distinguish the beneficial effects of PPARα and PPARδ signaling from ASCs during HF-like structure induction, the ASCs were pretreated with different PPAR agonists or antagonists for 2 days. The CM was collected and added to fresh DMEM to test ALP activities in DP cells (Fig. 5B). Only the addition of PPARα agonist in ASCs showed significant enhancement of ALP activity compared to the CM collected from ASCs (ASC 100% CM). Therefore, the core-shell structure of assembling ASCs surrounding the DP sphere can assist the structure arrangement as well as provide the correct molecular signaling to benefit the hair induction.

## Discussion

In the current study, we discovered several unique approaches for hair neogenesis. First, the DP characteristics can be preserved in 3D microenvironments by chitosan-coating surface. The quick loss of DP characteristics within a few passages of adherent DP cells highlights the importance of maintaining the 3D cluster during culture. To maintain the DP characteristics in culturing of adherent DP cells, several previous studies proposed various methods; the addition of Wnt3a, BMP6, or FGF2 to mouse DP cells^15^,^29^,^30^, the culture of rat DP cells with CM harvested from sole (palmoplantar) skin KC^31^, and the supplement of human DP cells with CM from neonatal foreskin KC^32^. However, these chemical approaches cannot reverse or prevent the loss DP markers *in vitro*. In addition, the loss of hair-inductive capacity in DP cells might be associated with the loss of aggregative behavior *in vivo*^33^. Therefore, the 3D culture system to form the sphere-like structure is essential for DP cells to maintain their HF-inducing capacity^14^,^15^,^16^,^17^,^19^. In our platform, the sphere size can be optimized and well controlled by seeding a specific density of DP cells onto the chitosan-coated 96-well plate.

Second, the assembling allows the further investigation of specific roles of ASCs in terms of *in vitro* spatial cellular arrangement. The CD34^+^ ASC was demonstrated to directly participate in HF morphogenesis by contributing to the formation of the dermal sheath which incorporates the whole HF, blood vessel, and adipose tissue^34^. To evaluate the effect of ASCs in interacting with DP cells, we applied ASCs to DPS via a mixture or a core-shell assembling method (Fig. 2). The protein expressions of Hey1 and VCAN in CSA-DPS were higher than MA-DPS which indicated the core-shell structure was better than the mixture structure for promoting hair-inductive capacity of DP cells *in vitro*. Freshly isolated DP cells from low passages are able to induce HF neogenesis in co-culture with epithelial keratinocytes^33^,^35^. The expression of VCAN is an important indication for HF induction which could help the DP sphere to interact with epidermal cells^15^. VCAN is a chondroitin sulfate proteoglycan and can serve as one of the major components of the extracellular matrix during HF morphogenesis, especially in the anagen phase of hair cycle^10^,^36^,^37^. Hey1, a signature gene for trichogenicity in DPs^15^,^30^, demonstrated an increase in expression during formation of the CSA-DPS (Fig. 2). On the other hand, the mixture of ASCs and DPs in MA-DPS approach might interrupt the direct cell-cell interaction and association in DP cells, or dilute the transmission of signals from ASCs to the DP sphere. The transplantation of CSA-DPS in the patch assay exhibited much more pigmentation and significant HF-like structure than the other groups which further confirm the importance of core-shell structure of CSA-DPS (Fig. 4). Our results demonstrated the participation of ASCs is important, especially with the consideration of spatial arrangements.

Third, different of adipose-related cells were well characterized from identical batches and the responses of DP cells in molecular and functional assessments were studied. Both the intrinsic molecular mechanism and the macroenvironment outside of HFs are important for activating the hair cycle and for regeneration. The intradermal white adipose tissue in the skin can communicate with HFs to regulate hair growth and regeneration. During the HF transition from telogen (rest) to anagen (growth), platelet-derived growth factor (PDGF) is known as one of the growth factors released by immature adipocytes, which surround the HFs, to trigger anagen activation^38^. The mature adipocytes also secrete leptin, adiponectin, and BMP2 to modulate hair growth^38^. ASCs can release PDGF, vascular endothelial growth factor (VEGF), transforming growth factor-beta (TGF-β), insulin-like growth factor (IGF), and hepatocyte growth factor (HGF)^39^,^40^,^41^ to induce tissue neovascularization and produce better microenvironments with abundant blood supply for tissues to regenerate HFs^42^. Application of CM from ASCs was shown to increase the proliferation of DP cells and keratinocytes through activation of proliferation related signals in HFs, such as Akt and ERK^43^,^44^. Modulation of PPAR signals are also important for skin tissue. PPAR agonists or antagonists may offer interesting opportunities for treating various skin disorders that caused by inflammation, cell hyper-proliferation, and abnormal differentiation^45^. PPARα is a master regulator of lipid metabolism and its expression is found in tissues that rapidly oxidize fatty acids, such as liver and brown adipose tissue. PPARα plays an important role in the formation of epidermal barrier and differentiation of sebocyte. PPARα is primarily activated through ligand binding which promotes KC differentiation. The addition of PPARα ligand, clofibrate, in culture increased the survival of human hair follicles^46^. However, some clinical reports found the direct uptake of PPARα-enhancing drug, Fenofibrate (Downlip®, 200 mg, 1 tablet per day), for controlling hyperlipidemia may have side-effects in skin to cause photoallergen with basal vacuolation and superficial perivascular inflammation^47^. Thus, the cell-based therapy to enhance local PPARα signal in CSA-DPS assembly as current study (Fig. 5) is a good strategy to improve the microenvironments for hair induction. PPARγ is involved in stimulating sebocyte development and lipogenesis. Although we found that adipocytes with high expression of PPARγ may not support the characteristics of DP spheres (Fig. 3), PPARγ signaling may still be required in HF stem cells which has been demonstrated that specific PPARγ deletion in HF stem cell causes scarring alopecia^48^. The stimulating PPARγ signal can effectively abrogate hair growth by premature catagen induction while protecting human HF epithelial stem cells^49^. Taken together, our current platform may provide a good approach to tune the PPAR signaling in specific adipose-related cells and study the interaction with DP spheres in HF formation.

The maintenance of the HF and its stem cells as a regeneration resource is important for optimal skin wound healing and hair formation^50^. Although the platform in current study provided several interesting ideas to promote hair induction, some improvements and applications can also be incorporated in future studies. We illustrated the core-shell structure of CSA-DPS is similar with the real condition for adipose tissue surrounding the DP of the HF. In physical HFs of normal skin, the core-shell structure is formed by keratinocytes wrapping up the DPs at the base of HFs. The usage of ASCs in current study can maintain and/or support the DP’s innate ability to induce hair after long term culture, possibly by reprogramming the cell state or by preventing differentiation (based on our *in vitro* results). The direct contact may be more efficient and may be necessary for future ‘hair engineering’ protocols. To further improve the ‘mimicking’ of the *in vivo* microenvironment, the multi-layer structures can be achieved by studying the interactions of DP sphere, KCs, and then ASCs from the inner core to the outer shell by using the assembling approach demonstrated in the current study. The enhancement of hair-inductive capacity was established by CSA-DPS assembling, but the detail interacting mechanism between ASCs and DP spheres is still not well understood particular in the paracrine and cell-cell association mechanisms. Specific inhibitors, blocking peptides, or siRNA can be introduced to the ASCs to alter the target signals responsible for activating the DP sphere and can be studied in the future including; VEGF, TGF-β, IGF, PDGF, and HGF pathways.

Chitosan is one of ideal biomaterial that is constructed by glucosaminoglycans and could provide a non-protein matrix for cell culture and tissue engineering^21^. Sphere formation can be observed after culturing hepatocytes, melanocytes, or ASCs on the chitosan-coated surface for 3 days^24^,^51^,^52^. The amino groups of chitosan can chelate calcium and form chitosan-calcium complexes to control the release of surface-bound calcium^53^. The decrease of surface-bound calcium levels can reduce the average diameter of ASC spheres^54^,^55^. The DP cells formed a spheroid-like structure on chitosan-coated surfaces with similar aggregation properties to ASCs. Therefore, the control of surface-bound calcium may also be considered to modify the process of chitosan for DP spheres. Other sphere formation methods, such as non-adhesion surface and hanging drop, can also be considered to promote the DP sphere formation. However, the non-adhesion surface may not induce cell reprogramming and the hanging drop approach is difficult to control the sphere size or form core-shell structure. The core-shell assembling approaches were tested by using thermos-responsive methylcellulose hydrogel to compose the cord-blood mesenchymal stem cells (at core region) and human umbilical vascular ECs (as outer shell) for functional vasculogenesis^56^. This indicates other biomaterials can also be considered to generate hair spheroids with a core-shell architecture. The chitosan-coated microenvironments promote the induction of DP spheres with specific enhancements of VCAN and Hey1 in both gene and protein expressions. The coating of chitosan in 96-well plate with optimized seeding density can deliver a well-controlled DP sphere with unified size and characteristics. The sequential assembling of DP sphere with ASCs creates the core-shell structure to further benefit the DP sphere and promote hair-like structure. The ASC, but not a mature adipocyte, is a good candidate to endorse hair formation when arranged in a specific spatial distribution surrounding the DP sphere. Taken together, this study established an assembling approach and microenvironment that has important clinical implications for the treatment of hair loss and the regeneration of hair follicles.

## Methods

### Isolation of cells

DP cells were isolated from rat vibrissal HFs as described in previous research^16^. All the animals were provided by the Animal Center at National Cheng Kung University (NCKU) with the approval of experimental procedures from the Institutional Animal Care and Use Committee (IACUC). The methods were used in accordance with IACUC approved guidelines. Briefly, the Sprague Dawley (SD) rats (6–8 weeks old) were sacrificed by an over-dose of carbon dioxide (CO_2_) and cheek skin specimens were dissected to isolate the vibrissal HFs using scissors and forceps. The hair bulbs were cut with scissors and incubated in collagenase type I solution (2.5 mg/ml, Invitrogen) at 37 °C for 3 hrs. The DP clusters were cultured in DMEM with 20% fetal bovine serum (FBS; Hyclone) and 1% penicillin and streptomycin (P/S; Invitrogen) for first 5 days, and then changed to DMEM with 10% FBS and 1% P/S from passage 2 to 8.

Isolation of ASCs from the rat adipose tissue was established in previous research^12^. Briefly, the ASCs from SD rats (6–8 weeks old) was harvested by collagenase digestion. To closely correlate the clinical application of ASCs in the current study, human ASCs were also obtained from raw lipoaspirates after liposuction from healthy donors with informed consent and with the approval of the Internal Review Board (IRB) of National Cheng Kung University Hospital (NCKUH). Methods were carried out in accordance with the approved guidelines. The culture and isolation of human and rat ASCs were prepared according to our previous established protocols^24^,^57^. To compare the induction effect of ASCs and mature adipocytes in hair induction, the adipogenic differentiation medium consisting of 0.5 mM isobutylmethylxanthine (IBMX, Sigma), 1 μM dexamethasone (Sigma), 10 μg/ml insulin (Sigma), and 1 μM indomethacin (Sigma) was applied to ASCs for inducing mature adipocytes from the same donor.

To visualize regeneration of HF, primary KCs were isolated from neonatal mice (C57BL/6, post-natal day 1) according to previous research^15^. In the isolation of keratinocytes, the skin fragments were digested with 0.5% dispase (Invitrogen) in Hank’s Balanced Salt Solution (HBSS; Invitrogen) and shook overnight at 4 °C to separate the epidermis from the dermis. The epidermis was cut into fine pieces, digested with 0.1% trypsin at 37 °C for 20 min, inactivated by DMEM, and then filtered through a 70-μm cell strainer (BD, USA). The cells were resuspended for use in the *in vivo* hair neogenesis experiments after centrifuge.

### Preparation of chitosan-coated microenvironments and assembling of spheres

The preparation of chitosan coating surface was described in our previous study^24^. The chitosan solution was added to a 10-cm TCPS dish or 96-well culture plate (Nunc) to cover the cell culture surface.

The interactions of DP cells with ASCs in the microenvironment during hair formation were investigated by using several assembling approaches. For sphere formation, the DP cells were seeded on chitosan-coated surface in a 10-cm TCPS dish with a seeding density of 1.3 × 10^6^ cells for 3 days. Free aggregation and random sizes of DP spheres may form on 10-cm TCPS dish. To unify and optimize sphere size, DP cells were seeded onto the chitosan-coated 96-well plate with seeding densities of 0.5 × 10^4^, 1 × 10^4^, 2 × 10^4^, 5 × 10^4^, and 1 × 10^5^ cells per well. To obtain a circular spheroid, a shaker with 130 rpm was used for 3 days after seeding cells in 96-well plate.

To study the interaction of adipose-related cells with DP spheres, either the ASCs or differentiated mature adipocytes were mixed within the DP sphere (MA-DPS) or formed a core-shell arrangement outside the DP sphere (CSA-DPS). For mixed spheres, the DP cells (5 × 10^4^ cells/well) were mixed with ASCs or adipocytes (2.5 × 10^4^ cells/well) and then seeded into the chitosan-coated 96-well plate for 3 days. To create core-shell assembling spheres, a sequential seeding method was developed to form the core sphere by seeding DP cells (5 × 10^4^ cells) in 96-well plate for 48`hrs and then adding the ASCs or adipocytes (2.5 × 10^4^ cells) to assemble the outer shell for additional 24 hrs.

### *In vivo* hair formation by patch assay

The patch assay was used to test the efficiency of hair formation among various assembled spheres in nude mice as previously mentioned^58^. Briefly, the 32 male nude mice (6–8 weeks old, BioLASCO, Taiwan) were anesthetized by isoflurane (Panion & BF biotech, Taiwan) and a small full-thickness wound on the lateral dorsal skin was created. The DP cells were mixed with KCs (2.5 × 10^6^ cells) for a total final volume of 200 μl in DMEM and then injected into the hypodermis area with a 200 μl pipette. The pipette tip was cut with a 1 mm diameter opening to assure the loading of spheres. The mice were divided into 5 groups to receive different DP and adipose-related cell mixtures: (1) DP cells: individual DP cells, (2) DP Sphere: DP spheres, (3) DP Sphere + ASC: DP spheres and suspending ASCs, (4) MA-DPS: mixed sphere for ASCs and DP cells (5) CSA-DPS: core-shell sphere for DP cell in core center and ASC on the outer layer. The skin was sutured after injection and then the full-thickness tissue was harvested after sacrifice of mice at 4 weeks after the implantation. To make sure the success of patch assay, the injection of KCs with neonatal epithelial cells (harvested from C57BL/6, post-natal day 1) was performed as positive control group.

### Measurements of DP characteristics and histological assessments of hair-like structure

The gene expressions of DP and mesenchyme markers in adherent DP cells and DP spheres were measured by reverse transcription-polymerase chain reaction (RT-PCR) as described in the previous study^24^. The primers for DP markers were designed by Primer3 for alkaline phosphatase (ALP), versican (VCAN), HEY1, bone morphogenetic protein 6 (BMP6), and Wnt5a (Table 1). Vimentin (VIM) was used as a mesenchymal marker. Markers for ASCs and PPAR family were assessed to distinguish ASCs from differentiated mature adipocytes. Quantification of these gene expressions were assessed by semi-quantitative fold changes and normalized to the control group and the house keeping gene GAPDH. To further confirm the appearance of DP markers, immunofluorescence staining of DP cells and spheres were performed with specific antibodies. Briefly, the cells and spheres were fixed with 4% paraformaldehyde (Sigma), and incubated with specific primary antibody at 4 °C overnight. The protein expressions of VCAN (1:100, Santa Cruz), HEY1 (1:250, Thermo), and VIM (1:250, Abcam) were measured in both adherent cells and DP spheres. In the assembling of DP spheres with adipose-relate cells, the ASCs or adipocyte were labeled with DiI (Invitrogen) for 30min to visualize their distribution in mixed or core-shell spheres. The spatial protein expressions and cellular arrangements were visualized by a spinning disc confocal microscopy (DSU IX81, Olympus) or a multi-photon confocal microscope (FV1000MPE, Olympus). The fluorescent images for each independent color were quantified by the gray-level of 32-bit mono-color image using ImageJ software.

To further understand the beneficial effects of adipose-related cells, conditional media (CM) were collected from the culture medium of ASCs and mature adipocytes in DMEM containing 2%FBS for 48 hours. The CM was used as 100% (100% CM) or mixed with fresh DMEM containing 2%FBS (1:1, as 50% CM/DMEM) to culture DP cells for 7 days. The *in vitro* ALP staining was performed by using FAST BCIP/NBT (5-bromo-4-chloro-3-indolyl phosphate/nitro blue tetrazolium) substrate kit (Sigma) in according to the manufacture’s protocols.

Several histological stainings were performed to observe the tissue morphology and protein expression patterns in *in vivo* hair induction samples^59^. To measure the ALP activity on whole mount skin tissue, ALP buffer (pH = 8.7, 100 mM Tris pH 9.5, 100 mM NaCl, 5 mM MgCl_2_, 0.1% Tween-20) was applied to the skin tissue at 37 °C for 60 mins and stained with ALP activity by using the ALP buffer containing 0.66% NBT and 0.33% BCIP (Promega) at 37 °C for 30 mins in the dark. The ALP reaction was stopped by applying stop buffer (pH = 8.0, 5 mM EDTA, 20 mM Tris) for 20 mins and then rinsing the skin tissue twice with PBS. The gross image of ALP activity in skin tissue was acquired by regular camera and visualized using an upright microscope (Olympus).

The hematoxylin and eosin (H&E) and immunohistochemistry (IHC) were applied to visualize the tissue structure and *in vivo* protein expressions after cell transplantation. Briefly, the skin tissues were embedded in paraffin, and then sectioned with 5 μm thickness^59^. The induction efficiency was evaluated the numbers of transplanted spheres in H&E staining. The diameter of each hair was measured from the histological images as captured by tissue scanning microscopy (BX51, Olympus). The specific protein expression patterns in skin tissue were detected by IHC staining with intermediate filament keratin 5 (K5, 1:200, Abcam), keratin 17 (K17, 1:200, Abcam), hair cortex Cytokeratin (AE13, 1:200, Santa Cruz), and Trichohyalin (AE15, 1:200, Abcam) antibodies. The PPAR expressions were detected by PPARα (1:500, Abcam), PPARγ (1:250, Cayman), PPARδ (1:100, Cayman).

### Statistics

All experiments were repeated at least for three times and the data are expressed as the mean ± SD. Statistical analysis was performed using a one-way analysis of variance (ANOVA) and the Scheffe post hoc test. Values of *p* < 0.05 were considered statistically significant.

## Additional Information

**How to cite this article**: Huang, C.-F. *et al*. Assembling Composite Dermal Papilla Spheres with Adipose-derived Stem Cells to Enhance Hair Follicle Induction. *Sci. Rep*. **6**, 26436; doi: 10.1038/srep26436 (2016).

## Supplementary Material

Supplementary Information
