# Corrigendum: Assembling Composite Dermal Papilla Spheres with Adipose-derived Stem Cells to Enhance Hair Follicle Induction

**DOI:** 10.1038/srep31257

**Published:** 2016-11-14

**Authors:** Chin-Fu Huang, Ya-Ju Chang, Yuan-Yu Hsueh, Chia-Wei Huang, Duo-Hsiang Wang, Tzu-Chieh Huang, Yi-Ting Wu, Fong-Chin Su, Michael Hughes, Cheng-Ming Chuong, Chia-Ching Wu

Scientific Reports 6: Article number: 2643610.1038/srep26436; published online: 05 23 2016; updated: 11 14 2016

The original version of this Article contained errors in the affiliations.

“Department of Cell Biology and Anatomy, National Cheng Kung University, Tainan, 701, Taiwan”

was incomplete, and now reads:

“Department of Cell Biology and Anatomy, College of Medicine, National Cheng Kung University, Tainan, Taiwan, ROC.”

In addition, the affiliation

“Institute of Clinical Medicine, National Cheng Kung University, Tainan, 701, Taiwan”

Now reads:

“Institute of Clinical Medicine, National Cheng Kung University Hospital, Tainan, 701, Taiwan”

The affiliation

“Department of Pathology, University of Southern California, California 90033, USA”

Now reads:

“Department of Pathology, University of Southern California, Los Angeles California 90033, USA”

These errors have now been corrected in the HTML and PDF versions of this Article.

