# Supplementary material for: Assembling Composite Dermal Papilla Spheres with Adipose-derived Stem Cells to Enhance Hair Follicle Induction
Source: Sci Rep. 2016 May 23;6:26436. doi: 10.1038/srep26436 (PMC4876394; doi:10.1038/srep26436)
Supplement: Supplementary Information [file srep26436-s1.pdf]

Assembling Composite Dermal Papilla Spheres with Adipose-derived Stem Cells to Enhance Hair Follicle Induction

Chin-Fu Huang[1§], Ya-Ju Chang[2§], Yuan-Yu Hsueh[3], Chia-Wei Huang[2], Duo-Hsiang Wang[3], Tzu-Chieh Huang[2], Yi-Ting Wu[3,4], Fong-Chin Su[1], Michael Huges[5,6, 7], Cheng-Ming Chuong[5,6,7], Chia-Ching Wu[1,2,4,5]

[1] Department of Biomedical Engineering, National Cheng Kung University

[2] Institute of Basic Medical Science, National Cheng Kung University

[3] Division of Plastic Surgery, National Cheng Kung University Hospital

[4] Department of Cell Biology and Anatomy, National Cheng Kung University

[5] International Research Center for Wound Regeneration and Repair, National Cheng Kung University

[6] Institute of Clinical Medicine, National Cheng Kung University

[7] Department of Pathology, University of Southern California

Contact information of corresponding author

Address: No. 1, University Rd., Tainan, Taiwan 701.

Tel: +886-6-2353535 ext 5327

Fax: +886-6-2093007

E-mail: joshccwu@mail.ncku.edu.tw

Footnotes: [§] co-first author

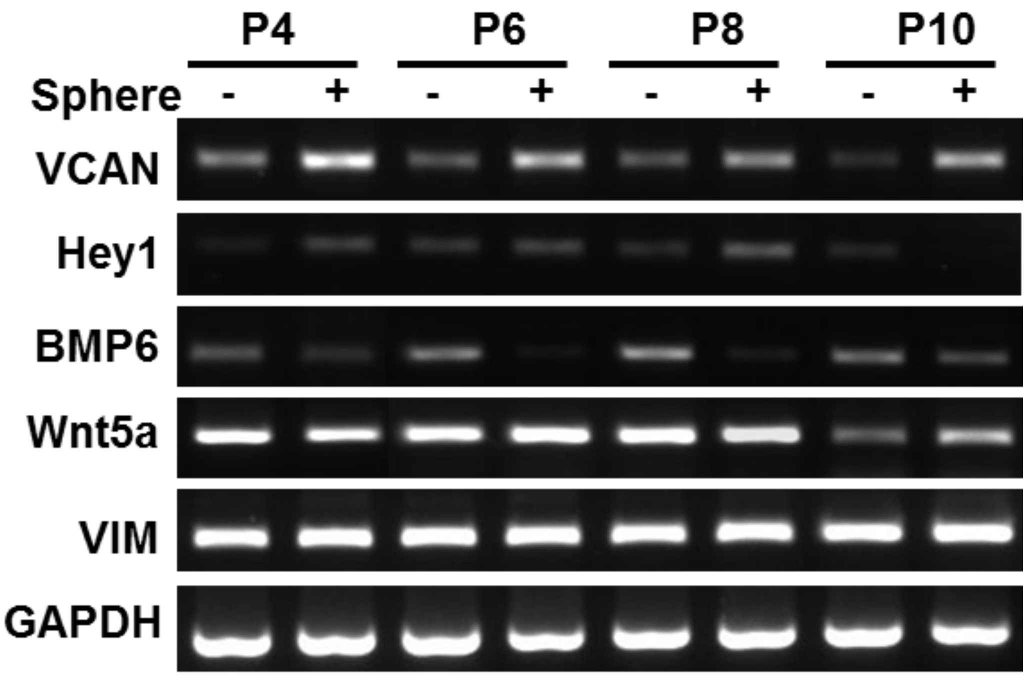

Supplementary Figure S1. DP cells isolated from rat were seeded on tissue culture polystyrene (TCPS) dish or chitosan-coated surface for sphere formation. Loss of DP characteristic, such as VCAN and Hey1, were observed in the adherent culture of DP cells. The sphere formation maintained the DP markers.

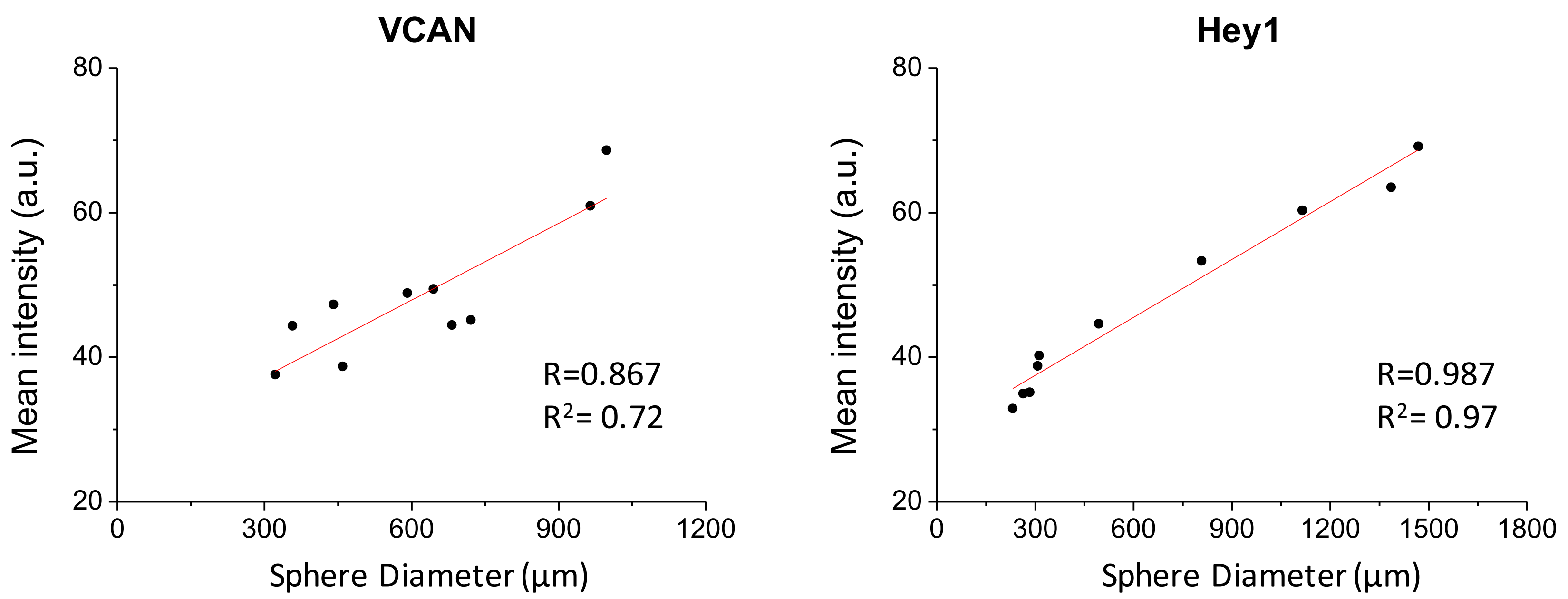

Supplementary Figure S2. The sphere size (x-axis) showed positive correlation with the quantified fluorescence intensity (y-axis) of VCAN and Hey1 staining.

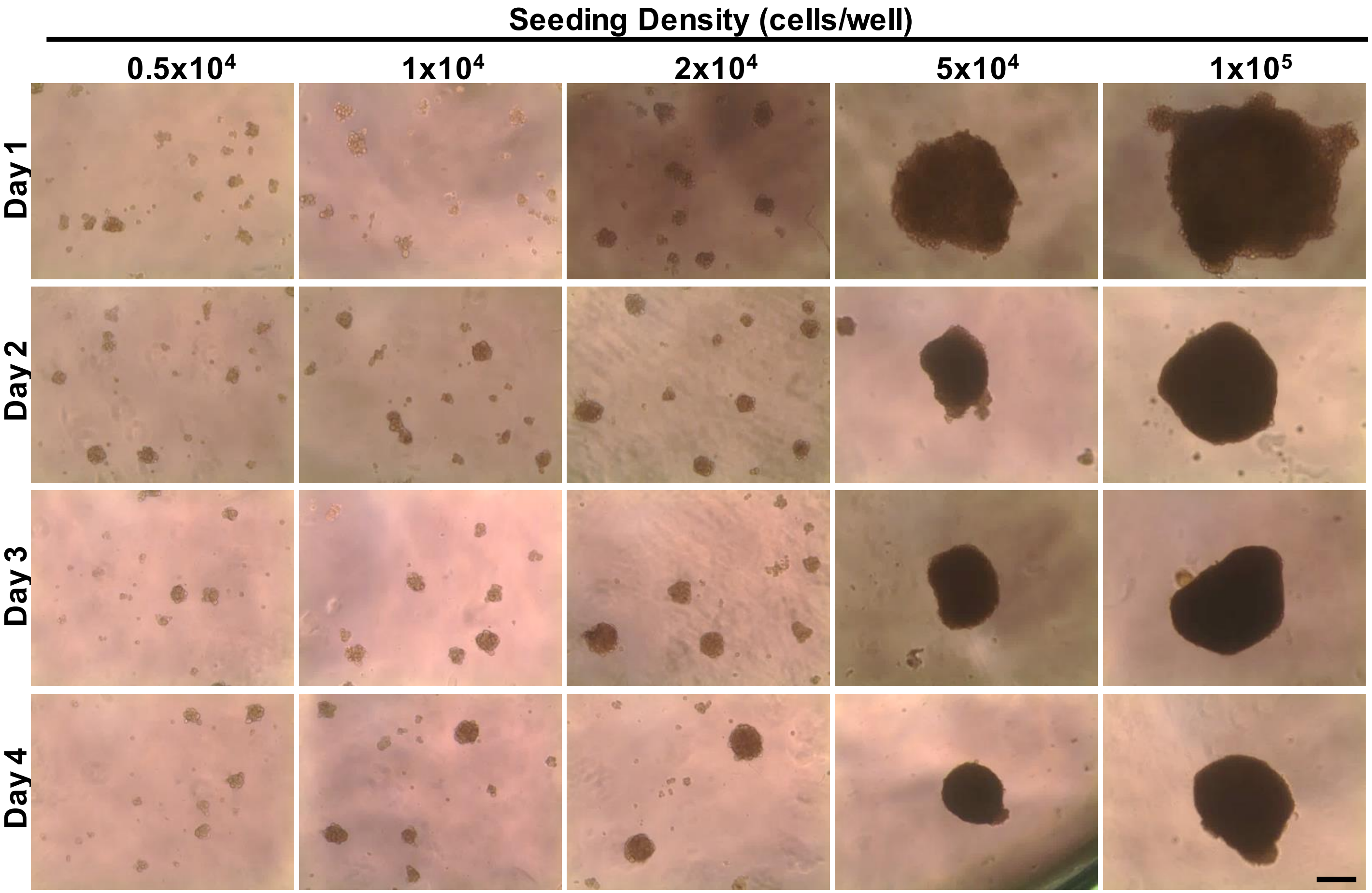

Supplementary Figure S3. Chitosan-coated microenvironments was created on the side walls and bottom of 96-well culture plate. The phase images were acquired to measure the DP sphere size with different cell seeding density for each well at Day 1, 2, 3, and 4.

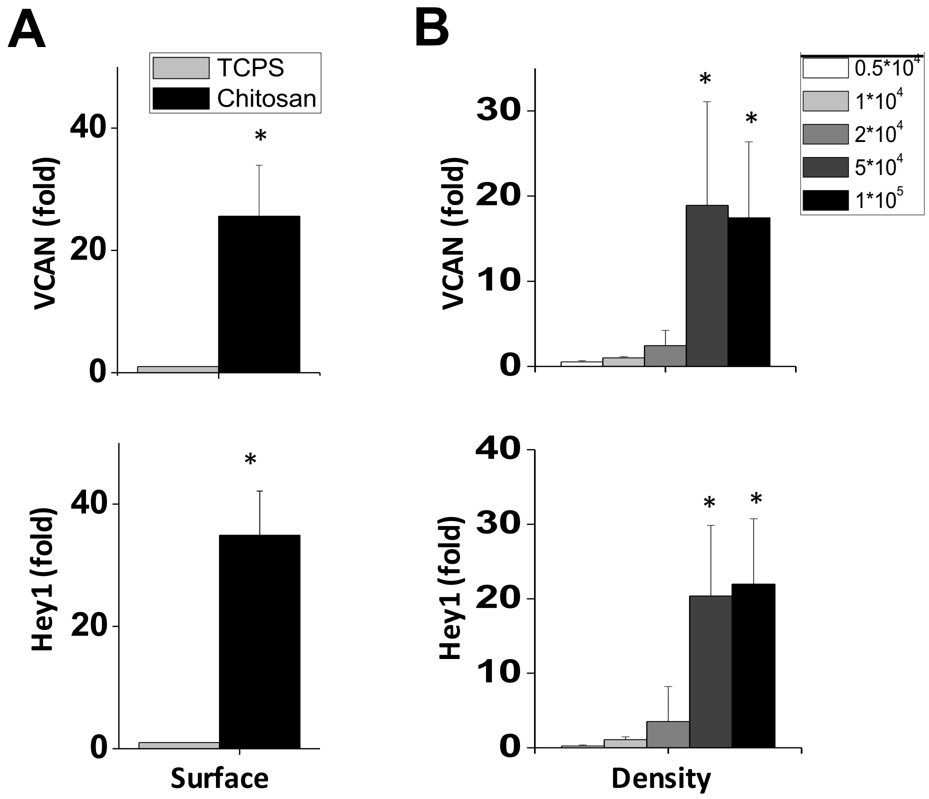

Supplementary Figure S4. Expression level of VCAN and Hey1 significant increased when DP cells were seeded in chitosan-coated TCPS (A). VCAN and Hey1 expressions were up-regulated as increasing the seeding density (B).

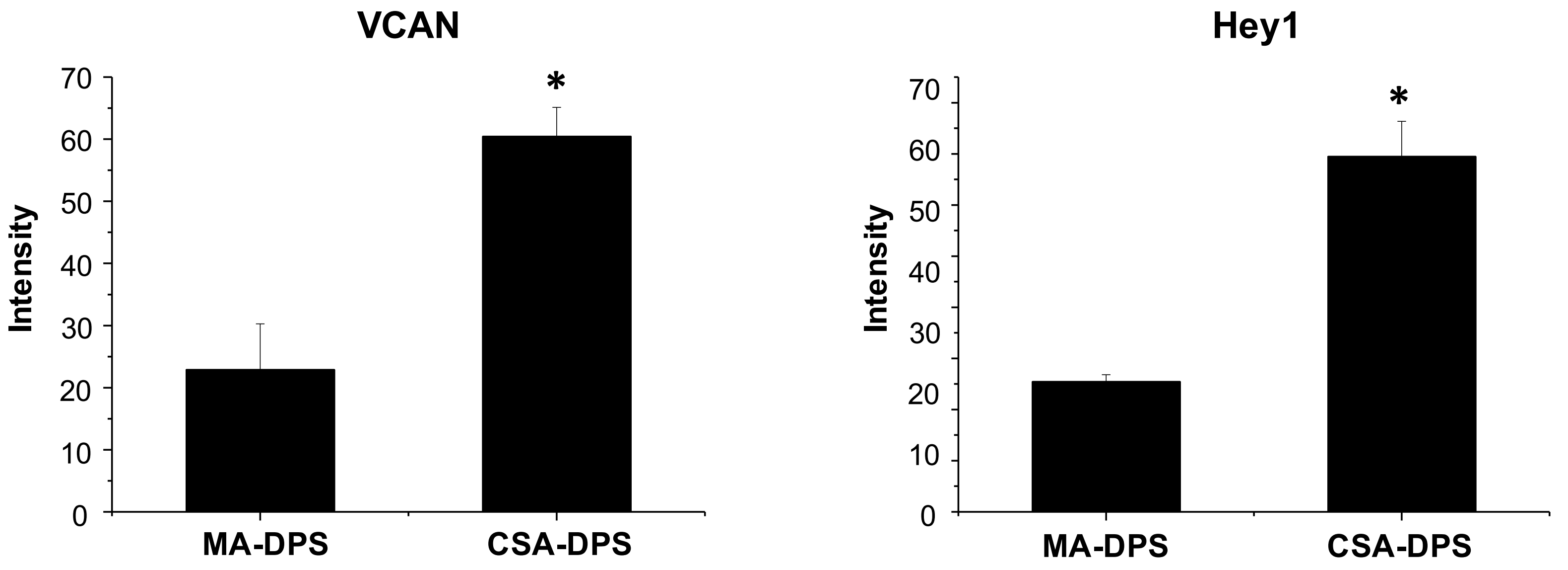

Supplementary Figure S5. The CSA-DPS significantly increased the protein expressions of VCAN and Hey1 as compared to the spheres formed by MA-DPS.

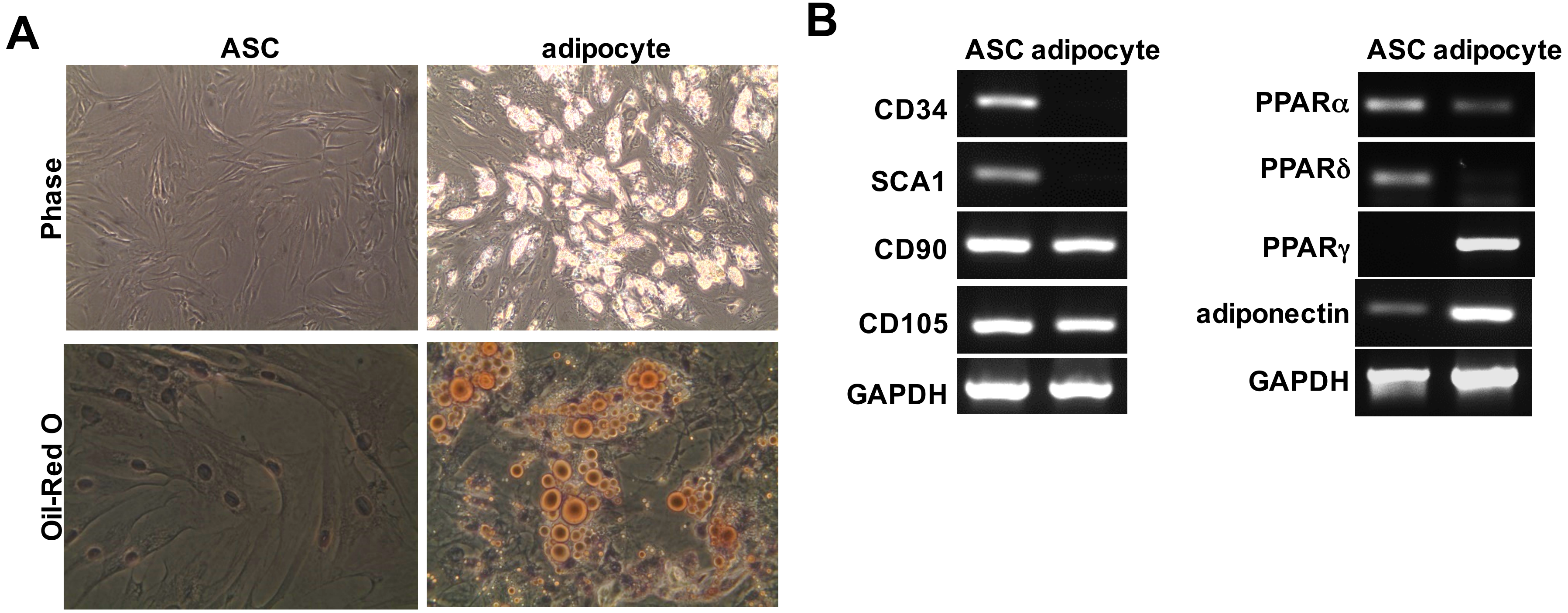

Supplementary Figure S6. The mature adipocyte differentiated from human ASC showed lipid droplets and positive staining of Oil-Red O staining (A). The ASC showed mesenchymal stem cell markers, the mature adipocyte decreased the expressions of CD34 and SCA1 (B). In addition, the adipose tissue markers for PPARr and adiponectin were elevated in mature adipocytes.

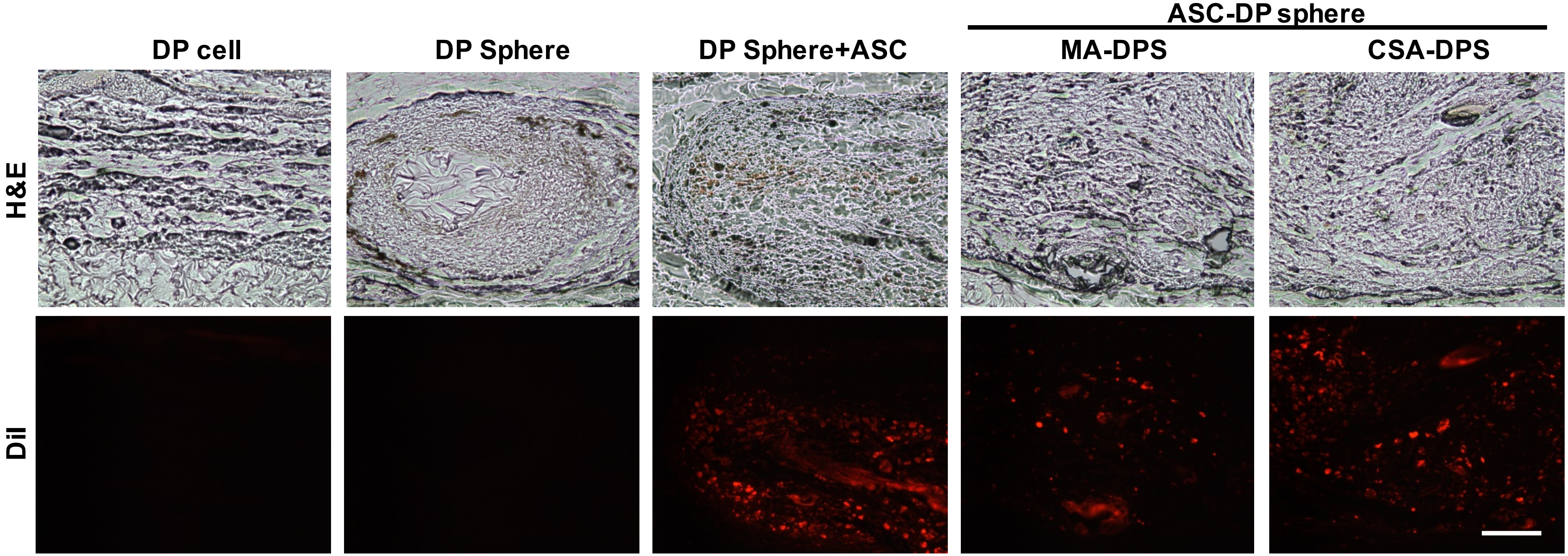

Supplementary Figure S7. DiI-labelled ASC appeared near HFs in skin patch. The transplantation of DP spheres with suspending ASCs (DP Sphere+ASC) showed increases of ASCs within the patch. The ASC-DP sphere showed specific location of these assembled DiI-labeled ASCs, especially in the CSA-DPS group.

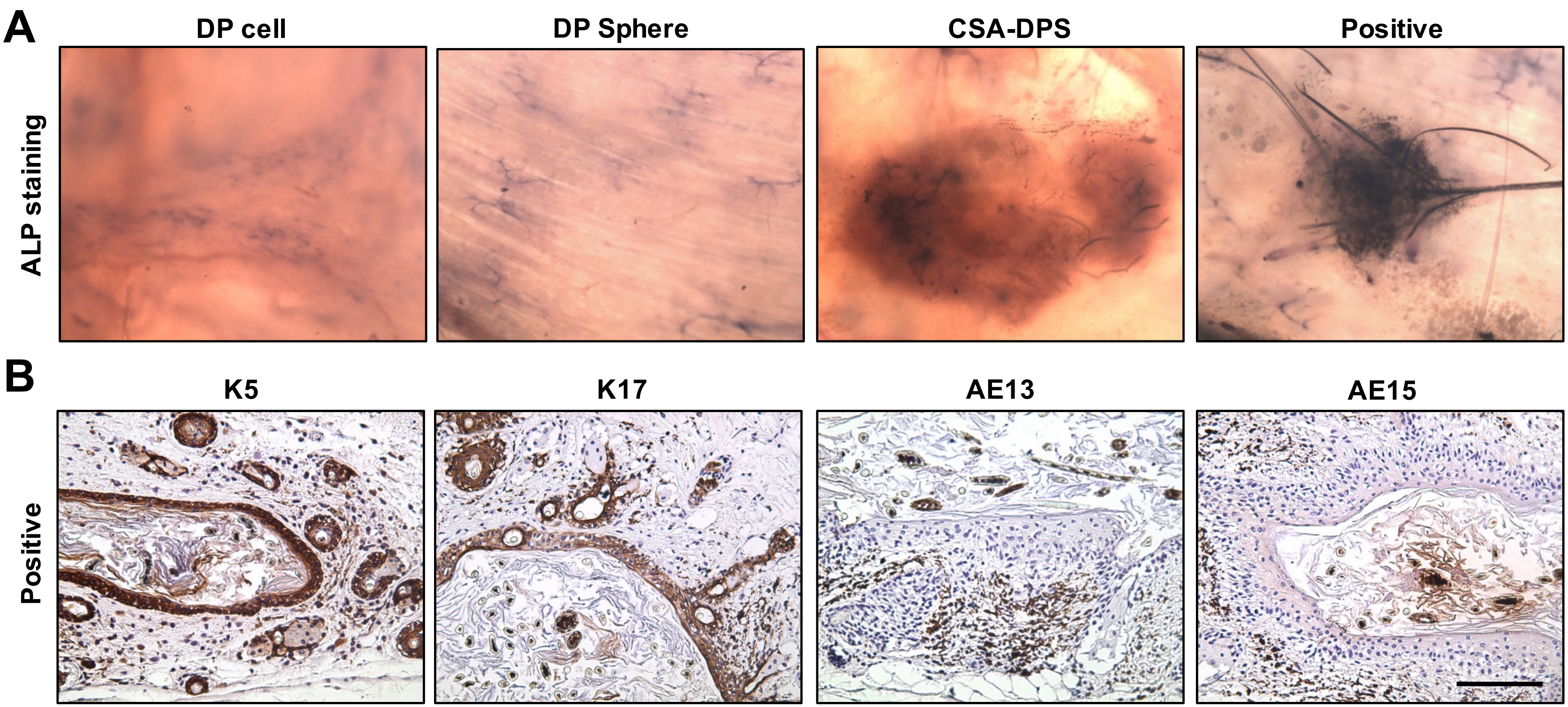

Supplementary Figure S8. Alkaline phosphatase (ALP) staining showed the ability of hair induction on CSA-DPS and positive groups (A). Positive staining of different HF structures in positive HF induction were observed by K5, K17, AE13, and AE15 markers (B). Although the relative HF induction in CSA-DPS is not as good as using neonatal cells (positive), the usage of adult DP cells and ASCs with CSA-DPS assembling still showed a significant improvement as compared to the transplantation of DP cells or DP spheres.

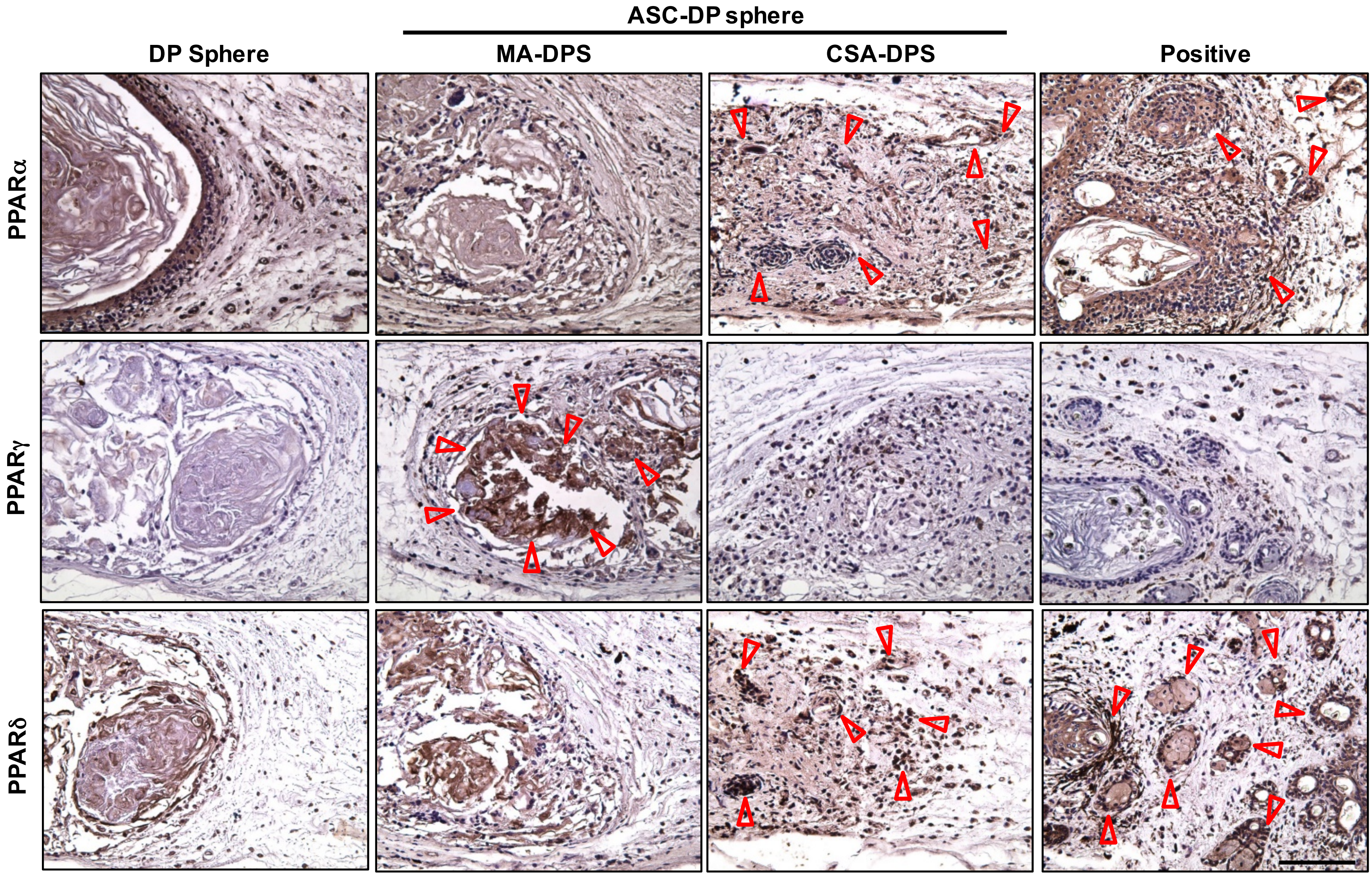

Supplementary Figure S9. Immunohistochemistry (IHC) stain showed highly expressions of PPARα and PPARδ near the HF in CSA-DPS and positive groups. This indicates the CSA-DPS assembling facilitates or preserves the ASCs to create the microenvironments with specific PPAR patterns that similar to the neonatal HF formations.
